# Cancer Evolution: Mathematical Models and Computational Inference

**DOI:** 10.1093/sysbio/syu081

**Published:** 2014-10-07

**Authors:** Niko Beerenwinkel, Roland F. Schwarz, Moritz Gerstung, Florian Markowetz

**Affiliations:** ^1^Department of Biosystems Science and Engineering, ETH Zurich, 4058 Basel, Switzerland; ^2^SIB Swiss Institute of Bioinformatics, 4058 Basel, Switzerland; ^3^European Molecular Biology Laboratory, European Bioinformatics Institute, Wellcome Trust Genome Campus, Hinxton, Cambridgeshire, CB10 1SA, United Kingdom; ^4^Wellcome Trust Sanger Institute, Hinxton, Cambridgeshire, CB10 1SA, United Kingdom; ^5^Cancer Research UK Cambridge Institute, University of Cambridge, Cambridge, CB20RE, United Kingdom

**Keywords:** Cancer, cancer progression, evolution, population genetics, probabilistic graphical models

## Abstract

Cancer is a somatic evolutionary process characterized by the accumulation of mutations, which contribute to tumor growth, clinical progression, immune escape, and drug resistance development. Evolutionary theory can be used to analyze the dynamics of tumor cell populations and to make inference about the evolutionary history of a tumor from molecular data. We review recent approaches to modeling the evolution of cancer, including population dynamics models of tumor initiation and progression, phylogenetic methods to model the evolutionary relationship between tumor subclones, and probabilistic graphical models to describe dependencies among mutations. Evolutionary modeling helps to understand how tumors arise and will also play an increasingly important prognostic role in predicting disease progression and the outcome of medical interventions, such as targeted therapy.

Cancer is a very heterogeneous disease. Genetic differences between people lead to differences in susceptibility (Pharoah et al. 2004), tumors develop in different organs and tissues of the body (Weinberg 2013), and cancers deriving from the same tissue can be stratified into disease subtypes based on differences in genomic measurements (Curtis et al. 2012). The genetic heterogeneity among cancer cells and the cellular heterogeneity of the tumor tissue underlie this phenotypic heterogeneity of the disease. The cancer cells in a tumor are not all identical, but form different clones, defined as sets of cancer cells that share a common genotype. Genetic and epigenetic heterogeneity poses a problem for the diagnosis and therapy of cancer. For example, it can lead to incorrect treatment decisions if a biopsy sample is not representative of other parts of the tumor (Merlo et al. 2006). In addition, tumor cells are part of the so-called tumor microenvironment, a heterogeneous tissue containing not only cancer cells, but also stromal and immune cells (Albini and Sporn 2007).

## 

### Cancer is an evolutionary process

Despite the heterogeneity of tumors, some functional organizing principles exist, often summarized as the hallmarks of cancer, which include evasion of apoptosis and immune response, unstable DNA, and the ability to metastasize (Hanahan and Weinberg 2000; 2011). A particular successful guide for understanding and modeling cancer progression has been evolutionary theory, which has a long tradition in cancer research. Already 40 years ago, seminal work established an evolutionary view of cancer (Nowell 1976; Dexter et al. 1978; Fidler 1978), in which carcinogenesis is regarded as an evolutionary process driven by stepwise somatic mutations and clonal expansions (Fig. 2a). Within each tumor, clones can evolve that harbor selectively advantageous mutations (called drivers), neutral mutations (called passengers), and deleterious mutations. The frequencies of passenger mutations can rise in a population by chance, often because they are linked to a driver mutation and hitchhike on the expanding clone. Some mutations increase the rate of other genetic changes and microenvironmental changes can also alter the fitness effects of mutations (Greaves and Maley 2012; Barcellos-Hoff et al. 2013). Moreover, there is evidence of competition, predation, parasitism, and mutualism between co-evolving clones in and around a tumor (Merlo et al. 2006). Most of these concepts were known for decades when advanced genomic technologies renewed interest in cancer evolution in the early 2000's. In the last few years, next-generation sequencing (NGS) of cancer genomes (Stratton et al. 2009) brought additional vigor to the field and has made tumor evolution a central topic in cancer research (Greaves and Maley 2012).

### Features of cancer evolution

Evolutionary theory is well developed and it provides an extensive toolkit for any evolutionary process. Modeling the somatic evolution of cancer also benefits greatly from this body of work. However, cancer evolution has several specific features, including the following four. (i) Cancer genomes harbor complex alterations. Many tumors display genetic instability, which results in abnormal numbers of chromosomes, that is aneuploidy (Weinberg 2013), elevated mutation rates, and altered distributions of mutational patterns (Garraway and Lander 2013). In addition, some genomic alterations can be extremely complex and rearrange entire chromosomes. These alterations enable large mutational jumps and they render comparisons between cancer genomes challenging. (ii) There are many selectively advantageous mutations. Recent cancer genome sequencing studies have identified hundreds of driver mutations (Vogelstein et al. 2013). Many of them disrupt cellular signaling pathways that are essential for multicellular organisms and may have occurred early in the evolution of multicellularity. These pathways tightly control and orchestrate cellular behavior in a tissue. In general, there are many ways to perturb a signaling pathway and hence, many possible mutations exist that increase the somatic fitness of cancer cells. In this sense, cancer evolution can be regarded as the evolution of defection (Nowak 2006a). (iii) Tumor cells are organized in specific structures. Population structure can result from interactions with the environment and from the spatial organization or the differentiation hierarchy of the tissue of origin. These structures affect the fate of mutations that occur in individual cells of the population, and hence the dynamics of tumor progression (Nowak et al. 2003). (iv) Tumorigenesis is a reproducible evolutionary process. Each tumor of the same type, or subtype, can be regarded as an independent realization of the same evolutionary process, even if some confounding factors will remain, for example, genetic background or microenvironment. Repeated observations provide an opportunity to enhance statistical inference about the evolution of tumors, which may eventually make cancer evolution more predictable (Orr 2005).

The evolutionary theory of cancer has survived 40 years of empirical observation and testing, and its components are well understood. However, at the same time, central questions remain controversial, for example, the argument of gradualism versus punctuated equilibrium, which is a long-standing debate also in species evolution (Gould and Eldredge 1993). The cancer community currently actively discusses whether tumors evolve gradually through a sequence of genetic alterations and clonal expansions that accumulate genomic lesions, or through a few punctuated changes driven by complex rearrangements (Baca et al. 2013; Shen 2013) or individual catastrophic events that shatter entire chromosomes (Stephens et al. 2011). In addition, large territories of cancer evolution still remain unexplored. For example, the evolutionary dynamics of cancer are still incompletely understood, because many parameters of this process have not yet been or can generally not be assessed experimentally, including fitness effects of mutations, generation times, population structure, the frequency of selective sweeps, and the selective effects of therapies (Merlo et al. 2006).

### Controlling cancer evolution

Research into cancer evolution not only addresses basic biological questions of tumor development and progression, but is also of clinical significance. For example, drug resistance is a major clinical problem resulting in therapeutic failure and uncontrolled disease progression. Even when patients initially respond well to cancer treatment, they often die because their tumors develop resistance to all available therapeutic avenues (Garraway and Jänne 2012). In his (1976) landmark article, Nowell wrote that “more research should be directed towards understanding and controlling the evolutionary process in tumors before it reaches the late stage seen in clinical cancer.” This statement is as true now as it was 40 years ago. A recent literature survey found that even though relapse and therapeutic resistance are inherently evolutionary processes, evolutionary concepts have not yet permeated cancer research (Aktipis et al. 2011), emphasizing the great need for evolutionary approaches to cancer biology and treatment.

Tumor heterogeneity and evolution as well as its clinical implications have been reviewed recently and extensively (de Bruin et al. 2013; Bedard et al. 2013; Klein 2013; Junttila and de Sauvage 2013; Burrell et al. 2013; Meacham and Morrison 2013; Almendro et al. 2013; Aparicio and Caldas 2013; Greaves and Maley 2012; Marusyk et al. 2012; Caldas 2012; Podlaha et al. 2012; Lambert et al. 2011; Michor and Polyak 2010; Salk et al. 2010; Bowtell 2010). In contrast to these biological and medical reviews, we focus here on mathematical and statistical methodology for modeling tumorigenesis. After reviewing the types of data available for modeling the evolution of cancer, we discuss several computational models, including population dynamics models of cancer cells, phylogenetic methods of tumor subclones, and probabilistic graphical models of tumor progression. Cancer-specific terminology used in this article is explained in Table 1.

**Table 1.** A table of common terms and acronyms used in cancer genomics

Term	Description
aCGH	Array CGH; microarray-based high-resolution CGH method
APC	Adenomatous polyposis coli; a human tumor suppressor gene (TSG)
Allele frequency	Fraction of cells (in NGS data, of reads) carrying a mutation
Aneuploidy	Abnormal number of (parts of) chromosomes (Fig. 1)
BAF	B allele frequency; ratio of the number of B alleles over the total (A+B) DNA content
BRAF	Gene coding for the B-Raf protein, a signal transduction kinase that can be involved in cancer if mutated
Carcinogenesis	The process of cancer development
Cellularity	The proportion of tumor cells in a sample
CGH	Comparative genome hybridization; cytogenetic method for analyzing copy number variations (CNVs)
Chromothripsis	The shattering of the genome in one catastrophic event (Fig. 1)
Chromoplexy	Chained rearrangement across several chromosomes (Fig. 1)
Clone	A set of tumor cells descending from the same ancestor and hence sharing its mutations
Clonal frequency	Percentage of tumor cells carrying an allele
CNV	Germline (normal) copy number variation in normal cells of the tissue and in tumor cells
CNA	Somatic copy number aberration (or alteration) in the cancer genome of tumor cells (Fig. 1)
Driver mutation	Mutation that confers a selective advantage
EGFR	Epidermal growth factor receptor; a cell-surface receptor whose altered expression is involved in cancer
Kataegis	Local hypermutation; many SNVs clustered on a short genomic segment (Fig. 1)
LOH	Loss of heterozygosity; Loss of one parental allele with or without a copy number change.
logR	Logarithmic intensity ratio of tumor and control DNA in an array CGH experiment.
NGS	Next-generation sequencing; High-throughput sequencing technologies based on massively parallel
	DNA amplification and sequencing
Oncogene	Gene that confers a selective advantage if hit by a gain-of-function mutation
Oncogenetic model	A model of the dependencies of events (CNAs, SNVs) in cancer development (Fig. 6)
Passenger mutation	Selectively neutral mutation
Phasing	The assignment of genomic alterations to specific haplotypes (Fig. 5)
Phylogenetic model	A model of the evolutionary relationship between different tumor samples from the same patient or between clones from the same tumor (Fig. 6)
Segmentation	The process of calling integer copy numbers from noisy logR values.
Somatic evolution	Evolution within an organism
SNP	Single nucleotide polymorphism; single base variant existing in the human population
	(found in normal tissue and tumor)
SNV	Single nucleotide variant; single base change that occurred in the tumor (Fig. 1)
TSG	Tumor suppressor gene; gene that confers a selective advantage if hit by a loss-of-function mutation
Tumorigenesis	The process of tumor development
Vascularization	The process of establishing blood vessels

## Molecular Cancer Data

The amount and the breadth of tumor molecular profiling has increased tremendously in recent years, mainly due to the advent of cost-effective high-throughput seqeuncing technologies. Genomic data on cancer stems from a variety of different sources, including (i) cell lines cultivated in laboratories, (ii) xenografts derived from patient tumors and engrafted into model organisms like mice, and (iii) clinical patient samples from biopsies. Experimentally, cell lines have several advantages over clinical samples: Initially they are genetically homogeneous, they can be kept under constant environmental conditions, and they show no contamination with normal cells. However, freed from its natural cellular context, this evolutionary process can have little in common with disease progression in patients, where, for example, the tissue microenvironment affects tumor evolution (Bissell and Hines 2011). Recent work on sequencing HeLa cells has shown that cancer cell lines can evolve to be very divergent and might be poor model systems for the disease they were derived from (Landry et al. 2013; Adey et al. 2013). Xenografts, although more closely related to a real tumor, are affected by different immune response and microenvironment in the host, but they can model tumor progression in a living organism over time with little sampling restrictions (Hidalgo et al. 2014).

Clinical samples are more direct reflections of the disease but incur logistic and scientific problems (Basik et al. 2013). For example, collecting multiple samples from patients can introduce biases, because patients with either very good or very poor response often contribute only few samples. Indeed, if the patient is cured after surgery or chemotherapy, no follow-up samples will be available, whereas in patients progressing very quickly, surgery is sometimes not attempted or the patient may succumb to the disease in short time. In addition, ethical and technical restrictions hinder broad collection of samples. A biopsy might be highly interesting from a scientific perspective but medically not necessary. Finally, clinical samples often suffer heavily from normal cell contamination (low cellularity) and infiltration of immune cells such as lymphocytes (Yuan et al. 2012).

### The Complexity of Cancer Genomes and the Search for Driver Genes

**Figure 1.**
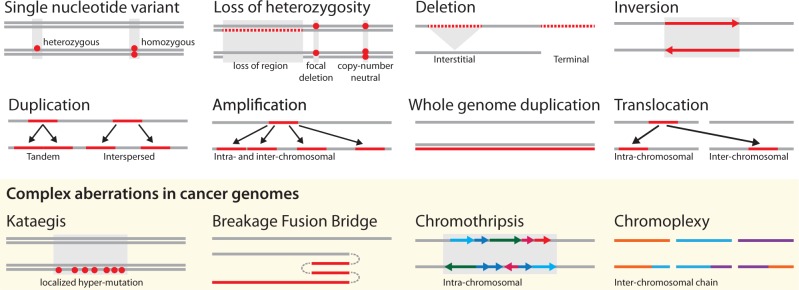
Common aberrations in cancer genomes. These events lead to the abnormal chromosome numbers (aneuploidy) and chromosome structures of a cancer genome. Lines indicate the genome with germline genome on top and cancer genome with somatic aberrations below. Double lines are used when differentiating heterozygous and homozygous changes is useful. Dots represent single nucleotide changes, whereas lines and arrows represent structural changes.

The normal human point mutation rate is $10^{-10}$ per base pair per cell division (Kunkel and Bebenek 2000), and this rate is elevated in many cancers, a phenomenon termed mutator phenotype (Loeb 2001; Loeb 2011). Hence, almost every cell division introduces a mutation, which inevitably leads to genetic diversity in every proliferating cell population. Cancer genomes are characterized by complex aberrations and rearrangements, ranging from small-scale point mutations (single-nucleotide variants; SNVs), often numbering in thousands per cancer cell, to large-scale chromosomal rearrangements resulting in complex patterns of genomic architecture and copy number aberrations (CNAs) (Greenman et al. 2012; Garraway and Lander 2013). Figure 1 gives an overview of genomic changes widespread in cancer. In addition, cancer genomes show epigenetic alterations, such as changes to DNA methylation (Hong et al. 2010; Sottoriva et al. 2013b). Some aberrations, like amplifications, deletions, and point mutations, are also common to many other evolutionary processes outside of cancer. Others, such as chromosomal deletions where one copy of a chromosome region is lost, are central to explanations of cancer evolution like the two-hit hypothesis (Nordling 1953).

In the following, we highlight complex patterns of aberrations that have recently been discovered in cancer genomes and whose evolutionary role is currently being discussed. *Kataegis* refers to a pattern of localized hypermutation, that is regional clustering of substitution mutations, observed in breast cancer genomes (Nik-Zainal et al. 2012a). *Breakage-fusion-bridge cycles* lead to palindromic genomic patterns, which can be an early step in DNA amplification (Guenthoer et al. 2012). *Chromothripsis* (chromosome shattering) refers to a single catastrophic event in which tens to hundreds of genomic rearrangements occur at the same time (Stephens et al. 2011). Although its exact cause is unclear, it is thought to be provoked by radiation exposure at a critical time point during cell cycle when chromosomes are condensed for mitosis. Cells that survive the catastrophe can have a selective advantage due to increased tumor cell growth, and their genomes often exhibit CNA patterns oscillating between one and two copies in the chromothriptic region. *Chromoplexy* is a process similar to chromothripsis in that it involves multiple genomic rearrangement events (Baca et al. 2013). The events often occur in a chain-like fashion connecting spatially distant areas of the genome that can affect multiple drivers from the same pathway at the same time despite their location on different chromosomes. Both chromothripsis and chromoplexy show random breakage and fusion of genomic segments, but several features set them apart: Chromothripsis displays hundreds of breakpoints clustered within a single chromosome, whereas rearrangements in chromoplexy are unclustered, usually number in the tens, and include multiple chromosomes (Shen 2013). Chromothripsis appears to be a single catastrophic event early in tumor progression, whereas chromoplexy can occur multiple times during cancer evolution and has been detected at the clonal and subclonal level (Baca et al. 2013).

The complexity of cancer genomes and the presence of mutator phenotypes make it challenging to separate driver from passenger mutations. To identify genes under positive somatic selection, one can detect an excess of nonsynonymous somatic mutations, that is, a high dN/dS ratio, in cancer genome sequences. The same genes are often under purifying selection in intergenerational terms leading to a depletion of nonsynonymous polymorphisms in the human population. Based on the idea of a high somatic dN/dS, (Greenman et al. (2006)) formulated a hypothesis test in a Poisson regression framework for discovering cancer driver genes, which was applied to identify 120 driver genes among 518 protein kinases in a cohort of 210 cancer samples (Greenman et al. 2007). More recent methods incorporate additional covariates, such as replication timing and gene expression data to refine estimates of the local mutation rate (Lawrence et al. 2013). Gonzalez-Perez et al. (2013) also accounted for the functional impact of mutations, as predicted, for example, by SIFT (Kumar et al. 2009) and PolyPhen2 (Adzhubei et al. 2010). In addition, they used evolutionary sequence conservation and clustering of mutations within each gene to identify driver genes. Recently, Lawrence et al. (2014) analyzed 4,742 cancers to present a list of 219 recurrently mutated cancer genes. As the authors suggest, this list may grow further in the future, as many driver genes are only infrequently mutated.

### Intratumor Heterogeneity and the Detection of Subclonal Alterations

It has long been known that tumors are composed of multiple cellular subpopulations with different genotypes (Nowell 1976), and modern genomic techniques have refined this observation (Burrell et al. 2013). Analyzing single cells is the most informative approach to assess the heterogeneity within a tumor. Cell sorting can be used to detect cellular phenotypic heterogeneity in blood cancers (Amir et al. 2013) and immunofluorescence *in situ* hybridization to highlight the genetic diversity of individual loci (Almendro et al. 2014). Progress in single-cell genomics (Shapiro et al. 2013) allows sequencing genomes of individual cells taken from a tumor (Navin et al. 2011; Hou et al. 2012; Xu et al. 2012; Potter et al. 2013). However, in most studies, the samples used are a mixture of cancer cells and stromal cells. In the following, we discuss how to analyze clonal architecture from genomic profiles of mixed samples.

Genomic data is typically obtained by NGS or by DNA microarrays. Sequencing has the advantage of being able to detect somatic SNVs as well as local tumor copy numbers by read depth analysis. By contrast, SNP arrays generally do not allow *de novo* discovery of SNVs, but the SNP probes allow for allele-specific copy number inference by considering bi-allelic frequency, that is, the ratio of the frequencies of the two parental alleles. The main objective when calling SNVs is to distinguish sequencing errors from true variants and separating germline from somatic changes. Algorithms solving this problem either employ frequentist statistical methods for modeling the distribution of variants per site in the genome, such as deepSNV (Gerstung et al. 2012), Varscan (Koboldt et al. 2012), and LoFreq (Wilm et al. 2012), or employ a Bayesian classifier framework, such as MuTect (Cibulskis et al. 2013).

Classical methods for CNA calling, called segmentation, are often ill-suited for cancer samples, because they do not take differences in cellularity nor changes in tumor ploidy into account. For those methods that do, segmentation broadly follows the same principles in most implementations: Normalized array intensities or normalized read counts are defined as the log ratio, logR, between the local DNA copy numbers in a mixture of cancer and normal cells and the average copy numbers in the mixture,

(1) $$logR=\frac{\alpha (n_{i}^{A}+n_{i}^{B})+2(1-\alpha )}{\alpha P+2(1-\alpha )}$$

Here, α is the cellularity, P is the average tumor ploidy to which array intensities and read counts are normalized, and $n_{i}^{A}$ and $n_{i}^{B}$ are the integer copy numbers of the two parental alleles at locus i. The B allele frequency (BAF)

(2) $$BAF=\frac{n_{i}^{B}}{n_{i}^{A}+n_{i}^{B}}$$

is the ratio between the B allele and the total allele count, which can be obtained from both sequencing and SNP arrays. It provides additional information about the copy number state at a certain genomic locus, as often only one allele is amplified or deleted.

Traditionally, cellularity was assessed by visual analysis of tumor cells, either manually by a pathologist or via image analysis (Yuan et al. 2012). Laser capture microdissection (Emmert-Buck et al. 1996) can be used to select more homogeneous areas from mixed tissue sections (Navin et al. 2010), but the procedure is time-consuming and generally only used in small studies. Most current methods for mixed samples estimate both cellularity and average tumor ploidy during segmentation, including PICNIC (Greenman et al. 2010), ABSOLUTE (Carter et al. 2012), and ASCAT (Van Loo et al. 2010). ASCAT calls copy numbers specifically for each allele, which results in two integer vectors of copy numbers, one for each parental allele. Because typically no linkage information between adjacent loci is available, at each site the larger of the two copy numbers, by convention, defines the major and the smaller the minor allele.

Unlike SNVs, where allelic frequencies can be directly derived from read counts, CNA calling is difficult in populations with subclonal structure, because the mixture of subclones leads to noninteger copy numbers which introduce deviations from the expected log-ratio (Equation 1) in array data or from the expected read counts in sequencing data.

To address this issue, Oesper et al. (2013) proposed THETA, an algorithm that infers the most likely collection of genomes and their proportions from NGS data, in the case where CNAs distinguish subpopulations. Nik-Zainal et al. (2012b) introduced the Battenberg algorithm (named after a checkered genomic pattern resembling Battenberg cake), which first assigns all SNPs to known haplotypes, a task known as phasing. Then it tests within haplotype blocks for small deviations of the BAF values (Equation 2) from those expected at normal diploid loci to assess whether there is sufficient evidence for a subclonal copy number change.

## Population Dynamics of Cancer Cells

Population genetics provides a well-developed mathematical theory of evolution (Durrett 2002; Ewens 2004), and many of these models and techniques have been applied to cancer. The most basic models assume no interactions among tumor cells and ignore any structure of the population. These strong simplifications result in mathematically tractable models that allow for calculating quantities of interest such as intratumor genetic diversity, the probability of fixation of a new mutant, or the age of the tumor. For this reason, models of well-mixed populations with constant selection are widely and successfully applied to the evolution of cancer, and they provide the starting point for more complex models. The population size, N, is an important parameter affecting not only the evolutionary dynamics of the population, but also the appropriate choice of the mathematical model. In small populations, allele sampling effects are more pronounced requiring stochastic modeling, whereas large populations often behave almost deterministically allowing for models based on differential equations.

Historically, multistage theory was the first approach to model tumor progression based only on cancer incidence data. Later, with the availability of protein and DNA sequence data, classical population genetics models of asexual populations have been applied to tumorigenesis, and several new models have been developed to address specific aspects of the somatic evolution of cancer. In this section, we first review classical multistage theory. Then, models are discussed that are either stochastic or deterministic and assume either a well-mixed or a structured population, followed by hybrid models. Lastly, we address modeling of cellular interactions using evolutionary games.

### Multistage Theory

**Figure 2.**
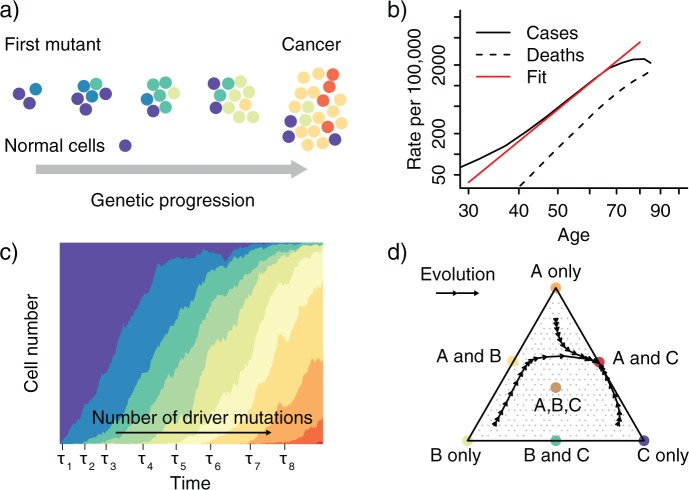
Modeling the population dynamics of cancer cells. a) Schematic illustration of the genetic progression from initially healthy cells (normal cells) to an invasive cancer by accumulating driver mutations. b) Age-incidence curves rise sharply above the age of 50 and are informative about the dynamics of tumor progression. The straight line shows a fit with power 4.8. The log-log-linear dependency of incidence on age is used in multistage theory to estimate the number of rate-limiting steps in cancer progression from incidence data. c) Population genetics models such as the Wright–Fisher model can be used to model the accumulation of driver mutations through multiple clonal expansions and to derive the average waiting times $\tau_{k}$ for a given number of alterations k. d) Dynamics of a three-strategy game corresponding to cell types A, B, and C. While simple additive fitness models always lead to the survival of the fittest, evolutionary game theory accounts for cellular interactions and allows for more complex dynamics, such as stable coexistence of cell types. Indicated here is a stable equilibrium with strategy A and C, but not B, which is reached from all three starting conditions via the indicated evolutionary paths.

Multistage theory models the probability of developing cancer as a function of age. The kinetics of tumor initiation and progression are usually unobserved, but different models can be tested and parameters can be inferred by fitting epidemiological age-incidence curves. In 1953, Nordling observed that cancer incidence rises sharply with higher ages and that it can be approximated by a monomial in age of degree six (Fig. 2b). Based on this observation, he postulated the existence of six independent rate-limiting steps during carcinogenesis. The underlying rationale of his inference was the following: If a single transforming step towards cancer occurs stochastically at a constant and small rate u, then the probability to observe this transition after time t is approximately ut. The cumulative probability of k successive steps is therefore proportional to $t^{k}$. Although the transforming steps occur stochastically in each patient, they will manifest on the population level, which allows the number of rate-limiting steps to be related to the observed age-incidence curves. Armitage and Doll (1954) repeated Nordling's analysis one year later and estimated that the exponent of the age-incidence curves ranged between 4.97 and 6.48 across a variety of cancer types. Using a similar reasoning, Knudson (1971) attributed the differences in the incidence of sporadic and hereditary retinoblastoma, a childhood cancer of the eye, to the existence of two independent rate-limiting mutations.

These early findings motivated the development of the so-called multistage theory of carcinogenesis (Frank 2007), which predicts that cancer incidence, I, defined as the first derivative of the cumulative number of cases, depends on age t as

(3) $$I(t)\propto u_{1}\cdots u_{k}t^{k-1}$$

where k is the number of stages and $u_{i}$ the transition rate from stage i−1 to i. Multistage theory has been extended to explicitly model the different kinetics of tumor initiation, namely the acquisition of the first transforming mutation in a renewing tissue, and the subsequent progression phases in the expanding tumor (Armitage and Doll 1957; Luebeck and Moolgavkar 2002; Calabrese et al. 2004; Meza et al. 2008; Luebeck et al. 2013). These stochastic models usually require fewer rate-limiting steps, as the rise in incidence is partly explained by exponential growth. Recently, multistage models have been used to optimize cancer screening strategies on the population level (Jeon et al. 2008; Dewanji et al. 2011).

The existence of up to six rate-limiting steps in cancer development has been widely accepted and appears to resemble the prevalence of driver mutations found in comprehensive cancer sequencing studies, where estimates range between two and eight drivers per tumor (Vogelstein et al. 2013).

### Stochastic Models of Well-mixed Populations

The assumption of a well-mixed cancer cell population, although questionable especially for solid tumors, is frequently made, and the mathematical approaches are best developed for this case. The most basic models assume constant population size.

#### Moran process and Wright–Fisher process

The Moran process and the Wright–Fisher process are the standard models for finite populations of constant size N. We assume two different cell types, referred to as normal and mutant (e.g., healthy and cancer cells, or two tumor cell types with different mutational patterns), with fitness $f_{1}$ and $f_{2}$, respectively. The Moran process defines a discrete-time Markov chain that keeps track of the number of mutants, X(t), in the population in generation t (Moran 1958). In each step of the process, a cell is randomly selected with probability proportional to its fitness to divide and produce one offspring, and another cell is selected for death uniformly at random. Each birth–death event leaves the total population size, N, unchanged and can alter the number of mutants, X(t), by at most one. The mutant subpopulation increases if a mutant is selected for reproduction and a normal cell for death. The probabilities of these two events are proportional to $f_{2}X(t)$ and N−X(t), respectively. The Moran process is the birth–death process defined by these transition probabilities. It has two absorbing states, namely extinction (X=0) and fixation (X=N) of the mutant.

In case of neutral evolution ($f_{1}=f_{2}$), the Moran process describes neutral drift of the population, that is, the fluctuation in allele frequencies due only to random offspring sampling Kimura 1983. In this setting, the probability of X mutants to reach fixation is x=X/N. If mutants have a selective advantage ($f_{1}<f_{2}$), then their fixation probability is

(4) $$\rho_{X}=\frac{1-{\left(f_{2}/f_{1}\right)}^{-X}}{1-{\left(f_{2}/f_{1}\right)}^{-N}}$$

and the mean time to fixation is ${\left(-2N\right)}^{2}[x\log x+(1-x)\log(1-x)]$ (Nowak 2006a). If u denotes the mutation rate, then the total mutation rate in a tumor of N cells is Nu and the rate of evolution, that is, the rate at which the entire population shifts from normal to mutant cells is $Nu\rho_{1}$. In the neutral case, the rate of evolution is equal to the mutation rate, because then $\rho_{1}=1/N$ (Kimura 1983).

The Wright–Fisher process is very similar to the Moran process, but in each generation, the entire population is drawn at random from the previous generation (Fisher 1930; Wright 1931). For two cell types and no selection, it is defined by the binomial sampling [X(t+1)∣X(t)]∼Binom(N, x(t)). Thus, cells are assumed to be synchronized and one generation in the Wright–Fisher process corresponds to N generations in the Moran process. With this rescaling, both processes have the same fixation probability, (essentially) the same fixation time, and the same diffusion limit, that is, they agree in the limit of large population size N→∞ (Ewens 2004). For computer simulations, the Wright–Fisher process generally allows drawing samples more efficiently.

When applied to cancer evolution, the Wright–Fisher process has been generalized to multiple cell types, representing genetically different tumor subclones, using multinomial sampling, and it has been extended to account for additional evolutionary forces, including mutation, selection, and genetic instability (Beerenwinkel et al. 2007a; Datta et al. 2013).

#### The coalescent

The coalescent is a stochastic process based on the Wright–Fisher process. It establishes a connection to observed sequence data by making inference about population parameters from a contemporary finite sample. In the coalescent, genealogies are generated by tracing coalescent events between lineages backwards in time (Kingman 1982). In the diffusion limit, N→∞, two lineages coalesce at rate $\left(\begin{array}{c} j\\ 2 \end{array}\right)$, when there are j individuals left (Rosenberg and Nordborg 2002). The coalescent accounts for mutations by imposing them on the random genealogy. It has originally been developed for neutral evolution, but has later been extended to account for selection (Neuhauser and Krone 1997).

The coalescent allows for inferring characteristic evolutionary parameters such as population size, mutation rate, or the time to the most recent common ancestor from sampled sequences. Nicolas et al. (2007) apply the coalescent to DNA methylation patterns of differentiated cells to estimate the number of stem cells in a human colonic crypt, that is, the number of cells at risk of initiating colon cancer. Statistical inference in the coalescent can be computationally demanding and several approximations to ML or Bayesian estimation have been proposed, most notably Approximate Bayesian Computation, which avoids evaluation of the likelihood function (Marjoram et al. 2003; Haccou et al. 2005).

#### Branching processes

Branching processes are well-studied stochastic models for populations of finite, but fluctuating, size (Athreya and Ney 1972; Kimmel and Axelrod 2002; Haccou et al. 2005). The basic assumption is that individuals produce a random number of offspring, each giving rise to an independent lineage that behaves identically to its parent, that is, after a certain lifetime, it will again produce a random number of offspring drawn from the same distribution. Most branching processes are inherently unstable: Eventually, the population will either die out or grow indefinitely. On the other hand, if a branching process with constant lifetime (i.e., a Galton–Watson process) and Poisson offspring distribution is conditioned on constant population size, one obtains the Wright–Fisher process (Haccou et al. 2005).

The probability generating function of the offspring distribution is the main mathematical tool for analyzing branching processes. It allows for computing several quantities of interest, including the extinction probability and time, and the probability of a mutant to arise and to establish its lineage in the population. For example, using a branching process with three different cell types, Danesh et al. (2012) have modeled ovarian cancer growth and progression, with the goal of identifying a window of opportunity for screening, that is, a time period during which tumor diagnosis is feasible (tumor large enough), but treatment still possible (tumor not progressed too far). Different tumor subclones can be modeled by multitype branching processes, where fitness advantages translate into altered offspring distributions. Because fitness parameters are generally unknown for cancer cells, Durrett et al. (2010) modeled them as random variables. They studied the effect of bounded versus unbounded fitness distributions on genetic tumor diversity and found that it depends only on the maximum attainable fitness advance, but not on the specific form of the fitness distribution (Durrett et al. 2011).

The development of resistance to targeted cancer treatment has been modeled by density-dependent branching processes. Here, tumor cell growth (and hence offspring distribution) is limited by tumor size due to geometric and metabolic constraints (Bozic et al. 2012). This modification removes the instability of the branching process and introduces a steady state in which the population size fluctuates around a constant value. The probability of tumor eradication under therapy can be computed in this model by considering the generation of resistance mutations during initial expansion, steady state, and treatment.

#### Diffusion approximation

Stochastic evolutionary models can be approximated by differential equation models to obtain simpler models that are more tractable and easier to interpret. The diffusion approximation is based on the assumption that the population size, N, is large and that the change per generation is small. It is given by the master equation, also called Kolmogorov forward, or Fokker–Planck equation (Fisher 1922; Kolmogorov 1931; Wright 1945; Feller 1951), for the probability density ψ(x,t) of the relative allele frequency x at time t in an evolutionary Markov process X(t), as

(5) $$\frac{\partial \psi (x,t)}{\partial t}=-\frac{\partial }{\partial x}M(x)\psi (x,t)+\frac{1}{2}\frac{\partial^{2}}{\partial x^{2}}V(x)\psi (x,t)$$

The second-order differential operator depends only on the mean M(x) and the variance V(x) of the Markov process X(t). This framework allows for analyzing or constructing evolutionary models with specific directional (M) and undirectional (V) forces. For example, at equilibrium of the diffusion limit (∂ψ/∂t=0), the Wright–Fisher process becomes

(6) $$\psi (x)\propto x^{\theta -1}{\left(1-x\right)}^{\theta -1}e^{\sigma x}$$

where θ=2Nu and σ=2Ns are the scaled mutation and selection parameters, respectively, and $s=f_{2}-f_{1}$ is the selective advantage of the fitter allele. This distribution reveals the strong impact of selection on large populations and of random genetic drift on small populations.

Tomasetti et al. (2013) used the diffusion approximation of the Moran process to calculate the expected number of passenger mutations that accumulate in the precancer phase to be BuT, where B is the total number of DNA bases sequenced, and T the number of times the tissue has self-renewed before tumor initiation. Using DNA sequencing data, they found that at least half of all somatic mutations in tumors of self-renewing tissues occur before the onset of neoplasia.

Diffusion theory is also useful for computing fixation probabilities and mean fixation times by considering the conditional density ψ(x,t|x(0)) (Ewens 2004). For example, in the Wright–Fisher process, the fixation probability of a new mutant in a haploid population is approximately 2s. The average fixation time is approximately 2Ns in the limit of weak selection, Ns≪1, and 2log(N−1)/s for strong selection (Otto and Whitlock 2001).

#### Tumor initiation and progression models

Specific evolutionary models have been proposed to describe the dynamics of tumor initiation and progression. The two basic genetic events driving carcinogenesis are gain-of-function mutations in oncogenes and loss-of-function mutations in tumor suppressor genes (TSGs). Oncogenes are activated by specific point mutations, gene amplification, or chromosomal fusion. In a well-mixed cell population of size N with constant mutation rate u, to activate the oncogene the fixation probability by time t is

(7) $$P(t)=1-\exp(-Nu\rho_{1}t)$$

where $Nu\rho_{1}$ is the rate of evolution (Michor et al. 2004). The equation shows that the accumulation of oncogene-activating mutations is faster in large than in small compartments. Thus, the organization of self-renewing tissues into many small compartments, such as, for example, the stem cell pools in colonic crypts, from which the tissue is derived, protects against cancer initiation.

The dynamics of TSG inactivation are more complex, because here two alleles need to be hit, either by two point mutations, or by one point mutation and loss of heterozygosity (LOH). For simplicity, we assume that the first hit does not confer any selective advantage. Then, in small populations and for short time spans t, the TSG fixation probability P(t) is quadratic in t, indicating two rate-limiting steps as in Knudson's two-hit theory. In intermediate populations, however, the second mutation may arise before the first one has reached fixation, a phenomenon termed stochastic tunneling. As a result, in intermediate populations, the TSG fixation probability is linear in t. Finally, there is no rate-limiting step in large populations (Nowak 2006a; Komarova 2007). Genetic instability can result in elevated mutation rates, which overall may or may not accelerate TSG inactivation, depending on the rate at which genetic instability is acquired and on the possible fitness costs it incurs.

The initial tumor cell lives in a hostile environment and generally has several possibilities to accumulate driver mutations to survive and expand. Using multitype branching processes, the evolutionary escape dynamics have been derived for arbitrary fitness landscapes defined on any genotype network, that is, any set of genotypes connected by mutation (Iwasa et al. 2003; Iwasa et al. 2004). Assuming that the initial tumor cell is too unfit to survive in the long run, the risk of escape is the probability of the population developing, before extinction, additional mutations that allow for escaping the selective pressure. For a given genotype network, the risk of escape is proportional to the per-site mutation rates and to the polynomial

(8) $$\sum_{i_{0}\to i_{1}\to \ldots \to i_{k}}f_{i_{1}}\cdots f_{i_{k-1}}$$

in the fitness values (Beerenwinkel et al. 2006). Here, the sum runs over all possible mutational pathways in the network from the initial and unfit genotype $i_{0}$ to the escape type $i_{k}$, where a mutational pathway is a sequence of viable mutants. The risk polynomial (Equation 8) captures the impact of the topology of genotype space on evolutionary escape. The larger the number of alternative escape pathways, the higher is the risk of escape. Studies on protein evolution have shown that only few mutational paths can lead to fitter proteins (Weinreich et al. 2006). Hence, understanding these mutational constraints for the evolutionary escape of cancer cells may make tumorigenesis more predictable.

During tumor progression, selectively advantageous mutations give rise to clonal expansions, which drive tumor growth. The series of selective sweeps is often described as a sequence of traveling mutant waves (Fig. 2c). To understand this process, mathematical models have been devised that address questions about the speed of evolution, the waiting time distribution, and the clone size distribution (Park et al. 2010). They are typically based on the Moran or the Wright–Fisher process, and different approximations have been proposed to arrive at the quantities of interest.

For example, assuming Wright–Fisher dynamics, the average waiting time for the first cell with k mutations to appear in a population of size N has been approximated as

(9) $$\tau_{k}=\frac{k}{2s\log N}{\left(\log\frac{s}{ud}\right)}^{2}$$

where d is the number of driver genes, each of which confers the selective advantage s (Beerenwinkel et al. 2007a). Thus, the waiting time is approximately linear in the number of mutations and tumor progression is driven mainly by selection. Schöllnberger et al. (2010) have identified the successive clonal expansions predicted by this model with the rate-limiting steps in multistage theory.

The rough approximation (Equation 9) has been refined and generalized in several ways. Using a branching process, Bozic et al. (2010) showed that in a growing population, the acquisition of subsequent driver mutations becomes increasingly faster. They estimated the average selective advantage per driver mutation from experimental data to be as small as s≈0.004. In addition, the stochasticity of the branching process provides an explanation for the huge heterogeneity in tumor sizes and progression times observed clinically. This model also allows for relating the expected number of driver mutations, k, to passenger mutations, n,

(10) $$n=\frac{\nu }{2s}\log\frac{4ks^{2}}{u^{2}}\log k$$

where ν is the rate of acquisition of neutral mutations, and u the driver mutation rate.

Durrett et al. (2009) considered a branching process approximation of the Moran process to arrive, in a rigorous analysis, at waiting times generalizing Equation 9 and the results for TSG inactivation (Durrett and Mayberry 2011). Furthermore, mutation accumulation has also been studied in exponentially growing cancer cell populations (Iwasa et al. 2006: Haeno et al. 2007; Durrett and Moseley 2010). McFarland et al. (2013) used a stochastic birth–death process to study the effect of moderately deleterious, rather than neutral, mutations on tumor progression and to explore cancer treatments that exploit their genetic load. In an attempt to calculate waiting times to cancer under mutational order constraints, Gerstung and Beerenwinkel (2010) considered conditionally independent Poisson waiting times for each mutation and derived the waiting time, $\tau_{k}$, accounting for all mutational pathways in genotype space.

#### Models for the evolution of drug resistance

The evolution of drug resistance is frequently observed during cancer treatment. For example, *BRAF* mutant melanomas treated by a targeted inhibitor often acquire resistance through mutations in other genes after initial response (Nazarian et al. 2010). A classical model for testing whether resistance is acquired during treatment or caused by pre-existing subclones is the model of Luria and Delbrück (1943). In their pioneering work on resistance of bacteria to infection with a bacteriophage, they calculated the relation between the mean and the variance of the number of drug resistant colonies emerging in an exponentially growing population. If resistance is acquired at the time of infection and cell-specific, then the number of resistant clones follows Poisson statistics, whereas the variance is larger if pre-existing resistant clones are selected, because resistant clones are not independent. Recently, Diaz Jr et al. (2012) used this model to conclude that in colorectal carcinomas, resistance to EGFR inhibitors, which is frequent and occurs after a rather constant time of treatment, is caused by pre-existing subclones. The analysis of Goldie and Coldman (1979) demonstrated that resistant subclones exist in a tumor with probability proportional to tumor size and mutation rate. Bozic et al. (2013) used branching processes to compute the probability of mono- and combination therapy success under the assumption that single mutations confer resistance to an individual drug and showed that combination therapy is much more likely to succeed.

### Stochastic Models of Structured Populations

The evolutionary dynamics of structured populations can differ from those in well-mixed populations. Population structure may result from cell differentiation into functional groups or from separation of cells into spatial compartments. For example, the simplest form of a regular grid is the n-dimensional lattice, where the number of interaction partners is identical for all cells. Komarova (2006, 2007) has shown that the fixation probability in the one-dimensional lattice is smaller than in a mixed population, because the number of interaction partners is constrained to the surrounding neighbors, which suppresses the effect of selection.

#### Linear systems

A simple one-dimensional system is motivated by the colonic crypt, which is organized as a vertical cylinder with a small number of stem cells at the bottom and the colonic epithelium at the top end. Stem cells divide slowly and produce differentiating cells that move up the cylinder until they reach the epithelium where they eventually shed off. Because of its radial symmetry, this process can be idealized by a linear chain of length N with a stem cell on one end and the epithelium on the other end (Nowak et al. 2003). As all but the stem cells are only transiently present in this system, only mutations in the stem cell will reach fixation, thereby reducing the number of cells at risk from N to just one. The process of shedding is regulated by the *APC* TSG, whose deactivation results in the accumulation of cells and their outgrowth as polyps. To inactivate both alleles of the tumor suppressor *APC*, the stem cell needs to be hit at least once followed by a second hit either in the stem cell or in a differentiating cell, as a double mutation in a differentiating cell is rather unlikely due to their short lifespan. The linear architecture of the colonic crypt can also be modeled by consecutive compartments of stem cells, differentiated cells, and transit cells, in which tumor growth is caused by imbalances between the rates of exchange between these (Johnston et al. 2007). Recently Zhao and Michor (2013) extended the linear process model to include spatially different growth kinetics along the crypt and found that the experimentally observed kinetics with an increased proliferation rate closer to the stem cell at the base of the crypt best suppressed the evolution of TSGs.

#### Cellular automata

The replication dynamics of cells on a discrete lattice in discrete time defines a cellular automaton (Deutsch and Moreira 2002). Each cell is represented by a separate object that stores the position, movement, and cell identity. The movement and replication dynamics are modeled by a set of rules accounting for the cellular state and its response to neighboring cells and microenvironmental stimuli. Cellular automata have the advantage that the fate of every cell is explicitly modeled. This imposes, however, a large computational cost when large ensembles of cells are simulated, and, in general, analytical results are infeasible.

Thalhauser et al. (2010) analyzed 1D and 2D spatial generalizations of the Moran process by including cell migration. They found that migration has a positive effect on the ability of a single mutant cell to invade a pre-existing colony and that large-scale cell death selects for the migratory phenotype. This finding may explain how chemotherapy provides a selection mechanism for highly invasive cancer cells.

Perfahl et al. (2011) proposed a complex 3D multiscale model of tumorigenesis that accounts for vascularization by coupling several processes, including blood flow, angiogenesis, nutrient and growth factor transport, cell movement, and interactions between normal and tumor cells. The agent-based model is analytically intractable, but in forward simulations, the spatio-temporal evolution of a vascular tumor can be investigated, including its response to therapy.

### Deterministic Models of Well-mixed Populations

In large well-mixed populations, stochastic effects can be negligible such that deterministic models of evolution based on dynamical systems can be applied that describe the mean behavior of the evolutionary system. Denoting by $x_{i}$ the frequency of genotype i and by ${\dot{x}}_{i}$ its derivative with respect to time, the replicator equation (Schuster and Sigmund 1983) is

(11) $${\dot{x}}_{i}=x_{i}[f_{i}(x)-\varphi (x)],\;\;i=1,\ldots ,n$$

where $f_{i}(x)=f_{i}(x_{1},\ldots ,x_{n})$ denotes the fitness which, in general, depends on the frequencies of all other genotypes. The term $\varphi (x)=\sum_{j}f_{j}(x)x_{j}$ is the average fitness of the population. In the special case of constant fitness, $f_{i}(x)=f_{i}$, the replicator equation is termed selection equation. In this case, the genotype with highest fitness reaches fixation, whereas all others go extinct; this is commonly referred to as survival of the fittest (Nowak 2006a). The selection equation can be extended to account for mutation with probability $q_{ij}$ from type i to type j to obtain the so-called quasispecies equation ${\dot{x}}_{i}=\sum_{j}x_{j}f_{j}q_{ji}-\varphi (x)x_{i}$. This model predicts an error threshold, that is, a critical mutation rate beyond which the population cannot be maintained due to loss of vital genetic information. Using this approach, Solé and Deisboeck (2004) explored the effect of genetic instability on tumor progression and found that an error threshold exists in mutator phenotype cancer cell populations, whereas Brumer et al. (2006) compared the effect of microsatellite and chromosomal instability on tumors.

### Deterministic Models of Structured Populations

At a macroscopic scale, the number of cancer cells in a given volume may be approximated by a continuous density. The continuum approximation of the dynamics on regular grids is given by partial differential equations (PDEs). A random spatial movement is then approximated as diffusion and a directed movement by physical drift (not to be confused with random genetic drift). The solutions of PDEs can be efficiently computed, and in certain limit cases, there exist analytical solutions or solutions that can be approximated by analytically tractable models. The latter approaches have the advantage that one can directly assess the influence of certain model parameters (Murray 2002). PDEs have been used to model the dynamics of tumor cell density and its dependency on other diffusive factors such as nutrients and growth factors. Many authors have studied avascular tumor growth to elucidate the role of nutrient flux and necrotic signaling during early tumor growth (Roose et al. 2007). Avascular tumor growth is characterized by an early exponential spherical expansion up to a diameter of about 1mm, above which cells on the inside become necrotic and growth saturates.

Population structure due to differentiation exists, for example, in the hierarchically organized hematopoietic system. It consists of a few thousand slowly replicating hematopoietic stem cells which differentiate in multiple steps into different mature blood cells. The evolutionary dynamics of the average number of cells in a given stage can be modeled by a system of ordinary differential equations. Such models show that the hierarchical organization suppresses the accumulation of mutations and correctly predict the clonal diversity in childhood acute lymphoblastic leukaemia (Werner et al. 2013). The different rates of cellular proliferation in stem cells and differentiating cells can also explain the biphasic response to imatinib treatment in chronic myeloid leukemia as well as the rates of relapse due to resistance mutations (Michor et al. 2005).

### Hybrid Models

Hybrid approaches of PDEs and cellular automata have been used to model the discrete dynamics of cell fates coupled to diffusive signals and nutrients fluxes (Anderson and Quaranta 2008). Dormann and Deutsch (2002) used a hybrid cellular automaton to model the avascular growth of tumor cells. Vascularization is a hallmark of cancer, and vascular endothelial growth factor (VEGF) signaling can be therapeutically targeted. Alarcón et al. (2005) used a multiscale model for the vascular tumor growth accounting for tumor-normal cell interactions and vascularization through VEGF signaling. Owen et al. (2009) used a similar model to understand the dynamics of vascularization under different drug pressures to optimize drug efficacy. A precise understanding of cancer growth kinetics may help optimize surgery and therapy duration, and eventually, quantitative models measuring the effect of drugs could be used for finding optimal drug dosage.

### Modeling Cellular Interactions using Evolutionary Games

Except for the replicator equation (Equation 11), all models discussed above assume constant fitness. However, the somatic fitness of cancer cells is likely to be density-dependent, because tumor cells interact with each other and with stromal cells (Poste et al. 1981). Evolutionary game theory provides a mathematical framework for modeling such interactions (Maynard Smith 1982). Here, fitness is the expected outcome of a game, which is defined by a payoff matrix. The dynamics of an evolutionary game can be either stochastic or deterministic and populations may be structured or not.

When two different cell types, or strategies, i and j meet and interact, then $M_{ij}$ denotes the payoff, that is, the benefit or harm, that cell type i receives from the interaction. In a well-mixed population, the expected payoff is a linear function of the population frequencies $x_{j}$, such that the fitness of type i becomes

(12) $$f_{i}(x)=\sum_{j}M_{ij}x_{j}$$

For infinite population size, the dynamics can be modeled by the replicator equation (Equation 11). The resulting dynamical behavior is much richer than in the situation with constant fitness, as it allows for multiple stable equilibria with coexistence of different cell types and for oscillatory patterns (Fig. 2d). In situations with multiple equilibria, the fixed point reached may also depend on the initial composition of the population. In the stochastic finite population size case, solutions of the corresponding Moran process have been derived in the limits of weak and strong selection (Taylor et al. 2004; Fudenberg et al. 2006).

A prototype two-strategy game offering insights into the evolution of cooperation and defection is the Prisoner's Dilemma (Axelrod and Hamilton 1981). In this game, cooperators pay a cost for others to achieve a benefit. Defectors, however, do not pay this cost, but nevertheless receive the benefit when playing against a cooperator. This leads to a situation where in the presence of co-operating cells a defector has a higher expected payoff leading to a selective advantage. The defecting strategy is the only evolutionarily stable strategy as it is a Nash equilibrium of the game. However, it does not maximize the overall population fitness as the payoff between two defectors is zero. The Prisoner's Dilemma game illustrates the somatic evolutionary advantage of defecting tumor cells in tissues of cooperative cells, which evolution generated in multicellular organisms (Nowak 2006a). It also raises the question under which conditions co-operativity can evolve. These conditions include different levels of reciprocity and group selection (Nowak 2006b).

Tumors consist of many cell types and interactions may occur at different levels. Vascularization is one hallmark of cancer, and tumor cells generally require the support of other stromal cells. Tumor cells also compete for resources, which introduces an interaction among them. Evolutionary game theory allows for modeling interactions between cancer and stromal cells and also cooperation among tumor cell types. Tomlinson (1997) has investigated the dynamics of multiple tumor cells where one cell type produces a substance cytotoxic to another and under which conditions a cytotoxic cell type can spread through the tumor. Gatenby and Vincent (2003) analyzed the evolution of cancer cells under growth control mechanisms competing for resources and found that such mechanisms can lead to multiple coexisting tumor cell types. Similarly, Axelrod et al. (2006) studied under which conditions cooperation among tumor cells can evolve. Gerstung et al. (2011b) investigated coexistence of multiple tumor types interacting with stromal cells using affine fitness functions, which include a constant fitness contribution in addition to the expected payoff. Due to the symmetry of the replicator equation, the corresponding replicator dynamics can be transformed to an equivalent game with different payoff (Stadler 1991). Other applications include the work by Dingli et al. (2009), who showed that the phenotypic variability commonly observed in multiple myeloma, a blood cancer, can be explained by an evolutionary game. Basanta et al. (2012) have modeled the dynamics of different prostate tumor strategies and their interactions with stromal tissue and have analyzed how the resulting dynamics can be influenced by therapy.

## Tumor Phylogeny

The reconstruction of evolutionary trees is a classical topic in computational and evolutionary biology with a wealth of algorithms and models of sequence evolution (Felsenstein 2003), and these methods can be applied to the somatic evolution of cancer (Fig. 6a). Cancer cells divide and accumulate mutations and genomic rearrangements to form clonal subpopulations, the taxa in the phylogenetic tree. Tumor phylogenies represent the evolutionary history of its subclones and can be used to test different hypotheses about tumor evolution (Navin and Hicks 2010). However, the specific features of tumor evolution and cancer data pose challenges to the direct application of classical phylogenetic models. For example, clinical samples contain an unknown number of novel cancer genomes with admixture of normal tissue, whereas classical phylogenetic approaches assume that taxa are known a priori. Additionally, short read NGS does not reveal complete haplotypes, but only individual alterations without information about their co-occurrence.

Many different types of data and cellular properties have been used for evolutionary analyses in cancer, including microsatellites (Frumkin et al. 2008) and lentiviral barcoding (Nolan-Stevaux et al. 2013), but in the following we mostly limit the discussion to SNVs and CNAs, two widely used data types (Gerlinger et al. 2012; Carlson et al. 2012). Inference from complex events like chromothripsis might become very important in the future, but currently only the basic concepts have been described and robust inference methods to identify these events are lacking (Korbel and Campbell 2013). We end the section with a short overview of the current developments in single-cell sequencing, which will provide new opportunities for tumor phylogeny reconstruction in the future. For a summary of the software discussed in this section, see Table 2.

**Table 2.** Software tools implementing phylogenetic methods for reconstructing within-patient and within-tumor evolutionary tumor histories.

Software	Data	Model / Inference	References
PhyloSub^a^	SNV	Tree-stick-breaking process, binomial / MCMC	(Adams et al. 2010)
PyClone^b^	SNV	Dirichlet Process, beta-binomial / MCMC	(Roth et al. 2014)
SciClone^c^	SNV	Beta mixture model	Miller et al. 2014
Clomial^d^	SNV	Binomial / EM	(Zare et al. 2014)
Trap^e^	SNV	Exhaustive search under constraints	(Strino et al. 2013)
CloneHD^f^	SNV + CNA	HMM, EM, Variational Bayes	(Fischer et al. 2014)
ThetA^g^	CNA	Maximum likelihood	(Oesper et al. 2013)
cancerTiming^h^	CNA	Maximum likelihood	(Purdom et al. 2013)
GRAFT^i^	CNA	Partial maximum likelihood	(Greenman et al. 2012)
MEDICC^j^	CNA	Finite state transducer, Minimum-event distance	(Schwarz et al. 2014b)
TuMult^k^	CNA	Breakpoint distance	(Letouzé et al. 2010)
TITAN^l^	CNA	HMM / EM	(Ha et al. 2014)

Notes: SNV, single-nucleotide variant; CNA, copy number aberration; MCMC, Markov-chain monte carlo; EM, expectation maximization; HMM, Hidden Markov Model;

^a^https://github.com/morrislab/phylosub

^b^http://compbio.bccrc.ca/software/pyclone

^c^https://github.com/genome/sciclone

^d^http://www.bioconductor.org/packages/devel/bioc/html/Clomial.html

^e^http://sourceforge.net/projects/klugerlab/files/TrAp

^f^https://github.com/andrej-fischer/cloneHD

^g^https://github.com/raphael-group/THetA

^h^http://cran.r-project.org/web/packages/cancerTiming

^i^http://www.sanger.ac.uk/genetics/CGP/Software/GRAFT

^j^https://bitbucket.org/rfs/medicc

^k^http://bioserv.rpbs.univ-paris-diderot.fr/ letouze/TuMult

^l^http://compbio.bccrc.ca/software/titan/

### Phylogenetic Tree Reconstruction from SNVs

**Figure 3.**
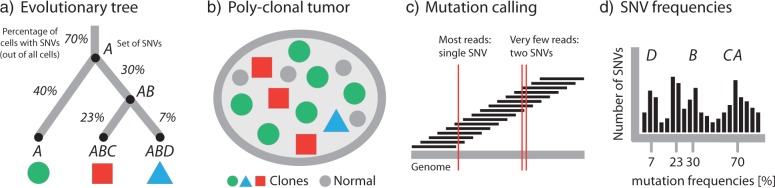
Inferring tumor phylogeny from next-generation sequencing data. a) Subclones are related to each other by an evolutionary process of acquisition of mutations. In this example, the three clones (leaf nodes) are characterized by different combinations of the four single nucleotide variant (SNV) sets A, B, C, and D. The percentages on the edges of the tree indicate the fraction of cells with this particular set of SNVs, e.g., 70% of all cells carry A, 40% additionally carry B, and only 7% carry A, B, and D. b) The evolutionary history of a tumor gives rise to a heterogeneous collection of normal cells (small discs) and cancer subclones (large discs, triangles, squares). Internal nodes that have been fully replaced by their descendants (like the one carrying SNV sets A and B without C or D) are no longer part of the tumor. c) Sequencing data consist of short reads covering (parts of) the cancer genome. Comparison to the germline DNA of the same patient allows to identify SNVs and other genomic aberrations. Since reads are short, most will only cover a single SNV. In few cases, pairs of SNVs are covered, which allows to assess patterns of co-occurrence and mutual exclusivity between SNVs. d) The sets of SNVs distinguishing the subclones cluster in the SNV frequency distribution. The mean of each cluster (x-axis) is the fraction of cells carrying this set of SNVs. The goal of tumor phylogenetics is to infer the evolutionary tree (a) from the mutations observed in the sequencing data (c) and their frequencies (d).

Sequencing a population of cancer cells allows to infer SNVs and their allele frequencies (Fig. 3). The allele frequencies need to be corrected for copy number alterations, LOH, and normal contamination to estimate the percentage of cancer cells carrying the SNV (Shah et al. 2012; Roth et al. 2014). Because it is unknown in which genome a given SNV occurred, prior to tree reconstruction, SNVs are clustered into sets of mutations with common frequency (Fig. 3d). This clustering is often performed using Bayesian mixture models, either finite ones (Larson and Fridley 2013) or nonparametric ones in which the number of mixture components is estimated together with their frequencies and densities (Shah et al. 2012; Nik-Zainal et al. 2012b; Roth et al. 2014).

**Figure 4.**
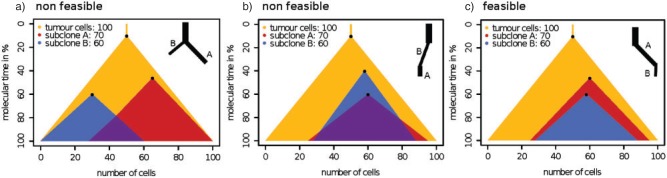
Two simple principles for tree inference from SNVs. For a given set of subclones and their respective clonal fractions, each illustrated by a triangle with a dot at the top vertex representing the clonal origin, two conditions need to be met for a potential phylogeny to be considered feasible: a) Dirichlet's box: If two SNV frequencies (small triangles inside large triangle) sum to more than 100%, then some cancer cells must contain both SNVs (overlap of the two small triangles). In a tree-like evolutionary process some cells must have acquired the same mutation independently, which in cancer, is considered highly unlikely. Hence, one of the two subclones (small triangles) is ancestral to the other. b) Larger ancestor: In this case, if one clonal fraction is larger than the other, the larger must be the ancestor; otherwise cancer cells would have lost the previously gained mutation (nonoverlapping regions between the two small triangles at the bottom), which again is considered highly unlikely. The most likely feasible solution is shown in c), where both principles are met (and the two small triangles are nested).

For manual phylogenetic tree reconstruction from inferred SNV frequencies, Nik-Zainal et al. (2012b) made two assumptions: (i) no mutation occurs twice in the course of cancer evolution (infinite sites assumption), and (ii) no mutation is ever lost (no back mutations). These assumptions translate into two basic principles: The pigeonhole principle or Dirichlet's Box (Fig. 4a), which in the simplest case states that if the sum of the clonal frequencies of two SNVs is greater than 100%, at least one cell must have contained both SNVs. Because the same mutation cannot be gained twice independently by the first assumption, one clone must be the ancestor of the other. The second assumption implies that the clone with the higher clonal frequency, that is, the larger number of cells carrying the SNV, must be the ancestor (Fig. 4b). These assumptions, although naive from a general phylogenetics perspective, appear to be justified in the cancer setting. Recent sequencing efforts showed that HeLa cells have accumulated about four million SNVs (Adey et al. 2013). Even with such a large number of mutations the probability of the same site being affected twice is about 1/1000, assuming a uniform mutation rate across the genome. Back mutations are accordingly less likely.

Computational methods have been proposed that implement these two rules. Strino et al. (2013) used a linear algebra approach that makes use of parsimony and sparsity assumptions to limit the number of possible trees. They show that their approach works for up to 25 aberrations, which makes extensive feature selection necessary, but it may be applied to the output of the mixture models discussed above by treating each cluster as a single aberration. However, Nik-Zainal et al. (2012b) demonstrated the limitations of sequential SNV clustering and tree reconstruction. In their analysis, one of the clusters was spread over three different branches of the tree. Such inconsistencies may be avoided by combining clustering and tree reconstruction into a single step. Recently, Zare et al. (2014) proposed a generalization of the approach implemented by Strino et al. (2013) to multiple samples per patient. Jiao et al. (2014) present a joint approach based on interleaving two stick-breaking processes, which results in a hierarchy of clusters (Adams et al. 2010). A recent implementation of a nonparametric Bayesian clustering approach that implements those principles is the work of Roth et al. (2014), which jointly infers posterior density estimates over both the clustering structure and cellular prevalence of the clones using MCMC sampling. An alternative is the work of Miller et al. (2014) which uses a variational Bayesian mixture model for subclone identification. Finally, Fischer et al. (2014) make use of the combined information in CNAs and SNVs to perform clonal decomposition using Hidden Markov Models.

It is important to note that single allele frequencies offer only a very limited snapshot of the tumor phylogeny. In particular, many different trees can agree with the same pattern of allele frequencies and only few topological constraints exist to limit the space of solutions (Fig. 4). Jiao et al. (2014) discuss this issue and find that SNV clusters can always be ordered into a linear cascade (with the most frequent one on top and the least frequent one at the bottom) and in a fork (unless the sum of frequencies of the child nodes is larger than the frequency of the parent node). Having multiple samples can further constrain the tree topology and, for example, predict a fork if the frequencies in the different samples put the clones into contradictory linear orders (Jiao et al. 2014). In summary, single allele frequencies offer only weak evidence for tumor phylogenies. Sequencing technologies with longer reads might alleviate these limitations in the near future, because more reads will carry multiple SNVs and patterns of co-occurance or mutual exclusivity will help to refine tree structure.

### Phylogenetic Tree Reconstruction from CNAs

In addition to SNVs, many cancers display a large amount of genomic rearrangements resulting in CNAs, which provide another source of data for inferring evolutionary relationships among cancer genomes. Copy number profiles are affected by the same challenges as SNVs, such as normal admixture and subclonal genomic changes. Attempts to address these issues include Sector Ploidy Profiling (Navin et al. 2010), which involves macrodissection of a physical sample into sectors followed by cell sorting according to total DNA content, which results in more genomically homogeneous cell populations. Copy number profiles were then computed by segmenting intensities derived from two-color aCGH microarrays. Trees were reconstructed using distances based on Pearson correlation between the logR values followed by neighbor-joining tree inference (Saitou and Nei 1987). The authors found that the breast cancers they studied could be divided into two groups, one genetically homogeneous group, called monogenomic tumors, and one genetically heterogeneous group, called polygenomic tumors.

Alternatively Oesper et al. (2013) have proposed a computational approach for subclonal decomposition from copy number profiles based on genome-wide segmented read depth information. In the same spirit Ha et al. (2014) implemented a Hidden Markov Model-based approach for identification of subclonal copy number profiles.

In any case, for tree reconstruction from copy number profiles the traditionally used Euclidean or correlation distances are ill-suited. Genomic sites affected by rearrangement events are not independent and identically distributed. By the nature of the DNA replication process all genomic rearrangement events have a specific start and end, duplicating or deleting all bases between those two loci (Hastings et al. 2009). This mechanism results in strong dependencies between adjacent loci that are not accounted for by Euclidean or correlation distances, because they consider all loci independently, rather than the actual events.

To address this limitation, Letouzé et al. (2010) proposed the TuMult method, which works on breakpoints, that is, loci at which the copy number changes, instead of full genomic profiles. Using a novel breakpoint distance they estimate the number of genomic events between two copy number profiles, and these distances are then used for tree reconstruction. TuMult has been applied by Sottoriva et al. (2013a) to estimating phylogenies of glioblastoma. Like earlier methods, *TuMult* does not take allele-specific copy numbers into account.

**Figure 5.**
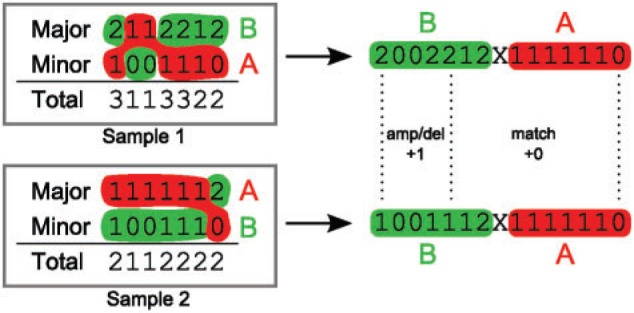
Phasing copy number profiles. While SNP arrays are capable of determining a major and minor copy number for the two parental alleles, their assignment (phasing) to the two actual physical alleles A and B is unknown. Because evolutionary events happen on the physical copies, correct phasing is essential for determining evolutionary distances. In this example, the two major copy number profiles between sample 1 and sample 2 (left) have a distance of two events (one amplification at position 1 and one amplification spanning positions 4 and 5), while the minor copy number profiles are identical, yielding a total of two events between the genomes of sample 1 and sample 2. Optimal assignment (right) to the alleles A and B reduces the evolutionary distance to a single amplification event spanning the first five genomic loci. This is also not evident from the total copy number (the sum of major and minor) which would still require two separate events.

Greenman et al. (2012) and Purdom et al. (2013) developed related algorithms for estimating the order of genomic rearrangement events. Although tree reconstruction is not the primary focus of these studies, ordering events involves solving similar problems as for estimating evolutionary distances. For example, for each site, the major and minor copy numbers must be assigned to one of the two physical alleles, that is, phased (Fig. 5). Greenman et al. (2012) use external linkage information that can, for example, be obtained from HapMap and a graph-theoretical approach to find the most likely assignment of each copy number to either of the two alleles. Ultimately, the method finds the most likely clonal ordering by solving this problem over all sampled genomes of a tumor. The phasing problem is solved in a similar fashion by the Battenberg algorithm (Nik-Zainal et al. 2012a).

Schwarz et al. (2014b) have recently developed MEDICC to jointly solve the problems of phasing and tree reconstruction using a minimum evolution criterion. Based on finite-state transducers (Cortes et al. 2004; Schwarz et al. 2010), MEDICC computes the minimum number of amplification and deletion events to transform one genomic profile into another. It finds allele-specific phasing, tree topology, and ancestral genomes such that the total tree length, that is, the total number of genomic events in the tree, is minimal. In an application of this approach to a large study of high-grade serous ovarian carcinoma, the authors confirmed the bi-partition of tumors into mono- and polygenomic cancers and showed that this stratification predicts resistance development (Schwarz et al. 2014a).

### Single-cell Approaches

For a small number of loci, fluorescent *in situ* hybridization has been used to characterize tumor heterogeneity on a single-cell level (Almendro et al. 2014; Trinh et al. 2014) and to infer phylogenetic trees (Chowdhury et al. 2013). On a genome-wide level, recent years have brought great advances in single-cell sequencing (Shapiro et al. 2013). For example, Navin et al. (2011) used low-coverage single-nucleus sequencing to reconstruct evolutionary histories of cancer lineages based on CNAs. They employed conventional neighbor-joining using a Euclidean distance metric on the discretized integer copy number profiles for tree building. Hou et al. (2012) demonstrated clonal evolution in essential thrombocythemia tumors by single-cell whole-exome sequencing of 90 individual cells and a population-level model of evolution, whereas Xu et al. (2012) found no evidence for clonal subpopulations when sequencing individual kidney cancer cells. One of the first statistical methods for evolutionary inference from single-cell sequencing data by Kim and Simon (2014) explicitly models the high single-cell sequencing error rates and infers phylogenetic relationships as well as orderings of mutations. Single-cell sequencing is still in its infancy and brings its own dedicated challenges, such as amplification bias, but has the potential to resolve some of the limitations of current approaches, most notably the phasing problem. In the near future, we expect most clinical studies on intratumor diversity to still rely on NGS of mixed samples, because of technical challenges and cost-benefit considerations.

## Cancer Progression

**Figure 6.**
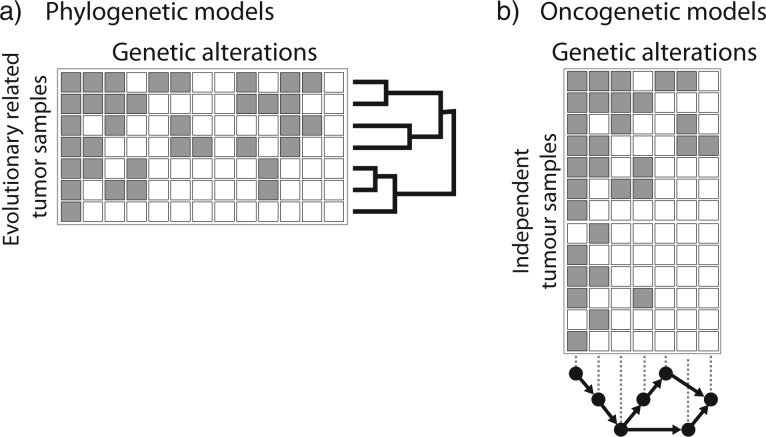
Phylogenetic versus oncogenetic models. Phylogenetic models of tumor samples (a) and oncogenetic models of cancer drivers (b) use the same type of data: genomic aberrations observed in patient tumor samples. Phylogenetic models (a) use mostly genomewide data of a small number of evolutionary-related tumor samples, either from the same patient or from different clones within the same tumor. Tumor progression models (b), on the other hand, generally concentrate on a small number of aberrations observed in a larger number of independent tumors from different patients.

Genetic events that drive cancer progression generally do not occur independently of each other. Direct and indirect interactions result from nonlinear, epistatic fitness landscapes underlying the evolutionary process and introduce statistical dependencies among genetic alterations. For example, Höglund et al. (2001) analyzed cytogenetic data from 3,016 solid cancers and observed preferential combinations of alterations using principal component analysis suggesting distinct evolutionary pathways. Graphical progression models are used to estimate these dependencies (Fig. 6b). In progression models, tumor samples from different patients are regarded as independent realizations of the same stochastic evolutionary process. The data is typically cross-sectional, that is, tumors are observed at different unknown time points. Most progression network models are variations of Bayesian networks, a class of directed graphical models representing conditional independencies among random variables. Available software for modeling cancer progression is summarized in Table 3.

**Table 3.** Software tools implementing probabilistic graphical models for estimating cancer progression.

Model	Topology	LPD	Constraints	Noise	Learning	Software	References
OT/HI	tree	discrete	monotone	no	ML	oncomodel^a^	(Desper et al. 2000;
							von Heydebreck et al. 2004)
OT	tree	discrete	monotone	no	MWB	oncotrees^b^	(Desper et al. 1999)
OT	tree	discrete	monotone	yes	MWB	oncotree^c^	(Szabo and Boucher 2002)
HOT	tree	discrete	monotone	yes	ML via SEM	n.a.	(Tofigh et al. 2011)
Mixture of OTs	forest	discrete	monotone	yes	ML via SEM	mtreemix^d^	(Beerenwinkel et al. 2005b;
							Beerenwinkel et al. 2005a)
Mixture of HOTs	forest	discrete	monotone	yes	ML via SEM	hotmix^e^	(Tofigh et al. 2011)
CBN	DAG	discrete	monotone	yes	ML	cbn^f^	(Beerenwinkel et al. 2007b)
CT-CBN	DAG	waiting time	monotone	no	ML via EM	ct-cbn^g^	(Beerenwinkel and Sullivant 2009)
Hidden CBN	DAG	waiting time	monotone	yes	ML via EM, SA	h-cbn^h^	(Gerstung et al. 2009)
Bayesian CBN	DAG	discrete	monotone	yes	MCMC	bayes-cbn^i^	(Sakoparnig and Beerenwinkel 2012)
NAM	DAG	waiting time	none	no	ML	n.a.	(Hjelm et al. 2006)
ON	DAG	discrete	(semi-)mon.	yes	MILP	diprog^j^	(Shahrabi Farahani and Lagergren 2013)
RESIC	none	RE	none	no	SM	upon request	(Attolini et al. 2010; Cheng et al. 2012)

Notes: OT, Oncogenetic tree; OT/HI, OT with hidden internal nodes; HOT, Hidden oncogenetic tree; CBN, Conjunctive Bayesian Network; CT-CBN, Continuous-time CBN; NAM, Network aberration model; ON, Oncogenetic network; LPD, local probability distribution; RESIC, Retracing the Evolutionary Steps in Cancer; DAG, directed acyclic graph; ML, maximum likelihood; EM, Expectation-Maximization algorithm; SEM, Structural EM; MCMC, Markov chain Monte Carlo; MWB, Maximum weight branching; MILP, mixed integer linear program; RE, Rate of evolution (Moran process); SA, Simulated annealing; SM, Simplex minimization by Nelder and Mead

^a^http://cran.r-project.org/web/packages/oncomodel/

^b^http://www.ncbi.nlm.nih.gov/CBBresearch/Schaffer/cgh.html

^c^http://cran.r-project.org/web/packages/Oncotree/

^d^http://mtreemix.bioinf.mpi-inf.mpg.de/

^e^https://github.com/atofigh/hotmix

^f^http://www.cbg.ethz.ch/software/cbn/

^g^http://www.cbg.ethz.ch/software/ct-cbn/

^h^http://www.cbg.ethz.ch/software/ct-cbn/

^i^http://www.cbg.ethz.ch/software/bayes-cbn/

^j^https://bitbucket.org/farahani/diprog

### Trees with Unobserved Internal Vertices

Among the first approaches to estimate dependencies among cancer-driving events were distance-based phylogenetic methods. The idea is to compute distances between genetic events, rather than between tumors as discussed above, and then to compute an optimal tree, where the observed genetic alterations are represented as the leaves of the tree. Desper et al. (2000) define the distance between events X and Y as

(13) −2logP(X,Y)+logP(X)+logP(Y)

which quantifies the deviation of the joint distribution P(X,Y) from P(X)P(Y), the one expected under the independence model. The authors subsequently apply distance-based tree reconstruction methods, such as neighbor-joining. von Heydebreck et al. (2004) have developed an efficient maximum likelihood approach for a probabilistic Bayesian network formulation of the same tree model, in which internal vertices correspond to hidden random variables. Although there is no obvious interpretation of the internal vertices, these models capture information about co-occurrences of events, and they can help detecting preferred sequential orders of events, specifically early events in tumor progression. Indeed, if an event X occurs almost always before event Y, then the leaf representing X will be close to the path from the root of the tree to vertex representing Y.

### Oncogenetic Trees

Oncogenetic tree models were introduced by Desper et al. (1999). They describe the accumulation of mutations under ordering constraints, which can be represented by a tree. The root of the tree is the wildtype without alterations. The branches at a given node describe the set of additional mutations that become possible when the node is mutated. Unlike the tree models discussed above, oncogenetic trees have no hidden internal vertices, but all vertices correspond to observed genetic alterations. The star defines the least restricted oncogenetic tree model, in which all alterations are possible at any time. The most restrictive topology is a linear chain of alterations, as in the case of sequential accumulation of *APC* → *KRAS* → *TP53* mutations during colorectal carcinogenesis, the first explicit description of cancer driver dependencies (Fearon and Vogelstein 1990). Desper et al. (1999) have proposed an efficient tree reconstruction algorithm based on an instance of maximum weight branching, a classical combinatorial optimization problem (Edmonds 1967; Karp 1971). Cancer progression models have been applied to CGH data, which records losses and gains of entire chromosome arms. For example, Jiang et al. (2000) used both distance-based and oncogenetic tree models to analyze dependencies among chromosomal aberrations in clear cell renal cell carcinoma. They found two distinct subgroups of tumors and clarified that loss of the small arm of chromosome 8 is a late event in renal cell carcinogenesis.

The original oncogenetic tree model from Desper et al. (1999) does not explicitly account for observation errors. Szabo and Boucher (2002) have made this extension to the model and the inference algorithm accounting for false positive and false negative observations. Oncogenetic trees have also been approached in the likelihood framework and extended to mixture models. Mixtures of oncogenetic trees provide a more flexible alternative to fitting a single tree, for example, in the presence of tumor subgroups, or alternative, mutually exclusive evolutionary pathways (Beerenwinkel et al. 2005a). They can be estimated by a structural EM algorithm, which, in each step, computes a maximum weight branching. To account for observation errors, a noise component with star topology is often used. Tree mixtures have been used to derive the genetic progression score, defined as the expected waiting time of the observed tumor in the model. This score improved survival predictions in glioblastoma and prostate cancers (Rahnenführer et al. 2005). Tofigh et al. (2011) have further generalized oncogenetic tree mixture models by introducing, for each event, a hidden random variable indicating a possible observation error. Their hidden-variable oncogenetic trees (HOTs) thus offer a different error model. Global structural EM algorithms for learning HOTs and mixtures of HOTs have been developed.

### Progression Networks

In tumor progression networks, the assumption of a tree-like dependency structure among alterations is dropped. General Bayesian network models have been proposed for this purpose (Radmacher et al. 2001), but they are often too computationally expensive to learn from data and not straightforward to interpret as progressions in time. A common assumption is monotonicity, that is, for each event, all its predecessors in the graph are required to occur before it can happen. For example, Conjunctive Bayesian networks (CBNs) are monotone progression networks. They generalize oncogenetic trees and are defined by a partially ordered set of mutations (Beerenwinkel et al. 2007b). In continuous-time CBNs, the waiting time for each mutation is assumed to be distributed exponentially; a genotype is defined by all mutations that have accumulated before a stopping time (Beerenwinkel and Sullivant 2009). Additionally, the observed genotypes may differ from the true genotypes because of observation errors (Gerstung et al. 2009). A nested EM algorithm is used to estimate the parameters of both the error and waiting time processes in the hidden CBN (H-CBN) model. The H-CBN allows for de-noising genotypes using the maximum a posteriori (MAP) estimates based on the progression model, which were found to improve survival predictions in renal cell carcinoma. Gerstung et al. (2011a) applied the H-CBN model to genetic data from three different cancer types, and found that there is stronger evidence for temporal dependencies among signaling pathways than among individual genes, likely because there are often many different ways to hit a signaling pathway. Using the Wright–Fisher model of cancer progression (Beerenwinkel et al. 2007a), they also showed that the accumulation rates of alterations in the CBN model are approximately linearly related to the fitness advantages s. Recently, a Bayesian inference scheme has been proposed for CBNs, which allows for assessing the full posterior probability of the partial order and the parameters (Sakoparnig and Beerenwinkel 2012).

Hjelm et al. (2006) have proposed an extended network aberration model (NAM), where aberrations are grouped together. Here, events not only occur randomly in time according to intensity parameters, but also the stopping intensity of the process after each event depends on the number of aberrations grouped into the event. In addition, the strength of all pairwise dependencies are explicitly parametrized in this model. A heuristic ML method is developed owing to the increased complexity of the model. More recently, progression network inference has been addressed using mixed integer linear programming. Shahrabi Farahani and Lagergren (2013) have devised and solved such an optimization problem for any decomposable model score, including the likelihood score and the Bayesian information criterion score. This approach works for monotone as well as for semimonotone networks, where only the presence of at least one predecessor mutation, rather than all, is required. Attolini et al. (2010) proposed a progression model in which the transition probabilities between genotypes are given by a Moran process. This method has then been extended by Cheng et al. (2012) to also account for transitions between cell states defined by altered signaling pathways.

All progression models discussed above aim at estimating the dependency structure among mutational events. Youn and Simon (2012) have proposed a statistical model for assessing the order of mutations without estimating their full dependency structure explicitly. Instead, they estimate the probability of each mutation i to occur as the k-th event in tumorigenesis directly. Although less informative about individual progression pathways, this approach may be more powerful for identifying early versus late mutations.

Sprouffske et al. (2011) used an agent-based model of a colon crypt to show that using cross-sectional data for inferring mutational order can be misleading. They emphasize the need for integrating phylogenetic methods based on intratumor samples to accurately reconstruct the evolutionary history of tumors. More generally, progression models will benefit greatly from assessing and resolving intrapatient and intratumor diversity. Integrating these two orthogonal modeling approaches (Fig. 6) is a major challenge for future work.

## Applications and Perspectives

Although cancer evolution is a fascinating topic for computational and evolutionary biologists, much of the progress in the field has so far been driven by the increasing integration of research into the clinic. On the other hand, already today, evolutionary modeling has an impact on the clinical management of cancer.

### Clinical Applications

Several clinical applications of evolutionary methods have been reported. Maley et al. (2006) showed that measures of clonal diversity can predict progression of the premalignant Barret's esophagus to a full-blown adenocarcinoma. In the future, this finding could enable clinical identification of high-risk patients that demand immediate treatment. Evolutionary studies can also give insight into the metastatic process and how selection pressure shapes the metastatic genotype. Khalique et al. (2009) demonstrated the clonal evolution of metastases from primary epithelial ovarian cancers using parsimony-based tree reconstruction on LOH events. In pancreatic cancer, a particularly aggressive malignancy, Campbell et al. (2010) identified genomic rearrangements that dysregulate the transition from the G1 to S phase of cell cycle and demonstrated convergent evolution among different metastases.

Complementary to studies that focus on early cancer development and the metastatic process, evolutionary studies have tried to identify sources of chemotherapy resistance. Cooke et al. (2011 showed that genetic heterogeneity indicates poor response to chemoradiotherapy in cervical cancer. In hereditary ovarian carcinomas, it was subsequently shown that secondary mutations that restore *BRCA1/2* predict chemotherapy resistance (Norquist et al. 2011). Later, an evolutionary study of high-grade serous ovarian cancers showed, for the first time, a correlation between genetic heterogeneity, patient survival, and chemotherapy resistance, and identified subclonal *NF1* deletions as potential drivers of resistant relapse (Schwarz et al. 2014a). Similarly, subclonal driver mutations have recently been identified that comprise risk factors for rapid disease progression in in chronic lymphocytic leukemia (Landau et al. 2013) and myelodysplasia (Papaemmanuil et al. 2013).

Evolutionary modeling can play an important role not only in supporting diagnostics and prognostics, but also for rationalizing treatment design (Bozic et al. 2012; Hochberg et al. 2013). For example, Chmielecki et al. (2011) use a 2-type branching process model with constraints derived from clinical data to predict dosing schedules of tyrosine kinase inhibitors against EGFR mutants. They found that the optimized dosing schemes may delay drug resistance development in lung cancer.

### Outlook

Recent advancements in high-throughput molecular profiling techniques allow for the assessment of the molecular states of tumors in great detail. Cancer genome data are collected at a large scale in many clinical studies and in international consortia, such as The Cancer Genome Atlas (TCGA) and the International Cancer Genome Consortium (ICGC). Most of these projects initially aim at characterizing the mutational landscape of various types of cancer by sequencing the tumors of many patients at low or moderate coverage (Stratton et al., 2009; Vogelstein et al., 2013). These data will be highly informative for discovering and cataloguing common aberrations in cancer genomes (Fig. 1) and for studying inter-tumor diversity and cancer progression (Fig. 6).

However, cancer is not only a disease of the genome, but also of abnormal cellular interactions in the tumor tissue. For example, the fitness of a clone depends on its genotype and the tissue environment the cells live in. The tissue microenvironment is a complex dynamical system with multiple cellular components that can influence cancer progression and evolution (Cairns 1975; Merlo et al. 2006; Lambert et al. 2011; Greaves and Maley 2012). Cancer cells are influenced by their tissue habitat, and reciprocally, they can remodel the tissue microenvironment to their competitive advantage (Barcellos-Hoff et al. 2013). Future studies will have to combine analyses of genetic heterogeneity with analyses of tumor tissue architecture to account for genetic variation and epigenetic variation of cancer cells as well as their interactions with surrounding cells (Marusyk et al. 2012; Barcellos-Hoff et al. 2013; Frank and Rosner 2012; Bissell and Hines 2011; Cairns 1975). Some progress is being made in this direction by jointly analyzing genomic data and pathological images (Yuan et al. 2012), but most evolutionary analyses are still performed on genetic data without any information of the tissue environment.

Once the technological hurdles of single-cell genomic profiling, such as inefficient and unbiased genome amplification, are overcome and individual cancer genomes can be identified reliably at a larger scale, tumor evolution can be studied more precisely and in greater detail. Novel and more powerful probabilistic models for these data will be required and are already being developed. They will need to take the spatial dynamics of tumors into account to allow for a systems view on cancer progression. Additionally, they will need to account for cancer-specific properties, such as generally nonhomogeneous rates of evolution. Cancer often requires deactivation of DNA repair pathways as an early driver event and in the course of clonal evolution, more of these events will follow, giving rise to mutator phenotypes. Other important questions concern cancer stem cells (Kreso and Dick 2014). Do all cancer cells have the capability of spawning new subclones and metastasize? Or is the metastatic potential limited to a small number of stem cells? The answers to these questions have important implications for the resulting tree topology, which could be fully branched, or star-like (Navin and Hicks 2010; Schwarz et al. 2014a).

Longitudinal sampling will pose both a challenge and an opportunity to phylogenetic reconstructions in cancer. Where in the traditional phylogenetics scenario all taxa are sampled at the same time point, different samples from biopsies before and after treatment might call for additional, more flexible evolutionary methodology. In this context, circulating tumor DNA has recently received a lot of attention. It has been shown that from tumor DNA found in the plasma of patients, tumor load, and genetic heterogeneity of the cancer can be inferred without the need for invasive biopsy or surgery (Forshew et al. 2012; Dawson et al. 2013; Murtaza et al. 2013). This opens many new possibilities for longitudinal sampling of patients that circumvent many of the inherent logistical and ethical complications of traditional clinical studies.

Intra-tumor genetic heterogeneity is often portrayed as a major challenge for successful targeted treatment. However, evolutionary analysis of the process leading to the observed heterogeneity could turn this perceived weakness into a strength by tailoring treatment specifically to the unique evolutionary scenario within each patient. Evolutionary models will thereby play an essential role in predicting escape mutations to treatment before they appear. Together with targeted therapy options, this approach will hopefully allow us to either ultimately eradicate the cancer or permanently restrict its growth, thus turning it into a chronic disease with low impact on quality of life.

## Funding

F.M. would like to acknowledge the support of The University of Cambridge, Cancer Research UK and Hutchison Whampoa Limited. Part of this work was funded by the European Research Council (ERC Synergy Grant No. 609883 to N.B.).
